# Interleukin-27 Signaling Promotes Immunity against Endogenously Arising Murine Tumors

**DOI:** 10.1371/journal.pone.0057469

**Published:** 2013-03-12

**Authors:** Karlo D. T. Natividad, Simon R. Junankar, Norhanani Mohd Redzwan, Radhika Nair, Rushika C. Wirasinha, Cecile King, Robert Brink, Alexander Swarbrick, Marcel Batten

**Affiliations:** 1 Immunological Diseases Division, Garvan Institute of Medical Research, Sydney, New South Wales, Australia; 2 Cancer Division, Garvan Institute of Medical Research, Sydney, New South Wales, Australia; 3 St. Vincent's Clinical School, University of New South Wales, Sydney, New South Wales, Australia; University of Palermo, Italy

## Abstract

Interleukin-27 (IL-27) is a pleiotropic cytokine but its immunosuppressive effects predominate during many *in vivo* immunological challenges. Despite this, evidence from tumor cell line transfer models suggested that IL-27 could promote immune responses in the tumor context. However, the role of IL-27 in immunity against tumors that develop *in situ* and in tumor immunosurveillance remain undefined. In this study, we demonstrate that tumor development and growth are accelerated in IL-27 receptor α *(Il27ra)*-deficient mice. Enhanced tumor growth in both carcinogen-induced fibrosarcoma and oncogene-driven mammary carcinoma was associated with decreased interferon-γ production by CD4 and CD8 T cells and increased numbers of regulatory T-cells (T_reg_). This is the first study to show that IL-27 promotes protective immune responses against endogenous tumors, which is critical as the basis for future development of an IL-27 based therapeutic agent.

## Introduction

Tumor immunosurveillance and anti-tumor immune responses are now appreciated to be an important mechanism for protection against the emergence and growth of tumors. The importance of immune mediated protection against cancer is demonstrated by the enhanced rates of tumor initiation and growth in immune compromised patients and mice [1]. However, it is clear that inflammation and antigen specific adaptive immunity can have both pro- and anti-tumorigenic effects [1], [2]. Thus, the type of response elicited is critical in determining the outcomes for tumor growth. In general, a T helper Type 1 (TH1) response, with accompanying IFN-γ production and cytotoxic T and NK cell activation, is associated with effective anti-tumor immune activity while TH2 and TH17 responses are pro-tumorigenic [2]. Regulatory T cells (T_reg_) actively inhibit anti-tumor responses and high levels of T_reg_ cells in patients correlate with poor prognosis in multiple cancer types [3], [4]. Thus, therapeutics that enhance TH1 responses or inhibit T_reg_ cell activity are actively being sought for cancer treatment.

IL-27 is a heterodimeric cytokine (IL-27p28:Ebi3) expressed by activated antigen presenting cells, which has sequence and structural similarity to IL-6, IL-23 and IL-12 [5]. IL-27 signals through a heterodimeric receptor, consisting of gp130 and IL-27rα, that is expressed by most hematopoietic cells [5], [6]. However, it is the regulation of T helper cell differentiation and function that appears to be the major biological function of IL-27. Analogous to IL-12, IL-27 promotes the differentiation of TH1 cells through activation of STAT1, induction of T-bet and the synthesis of IFN-γ [7], [8]. Meanwhile, it suppresses TH2 and TH17 differentiation [9], [10]. We and others have found that IL-27 can also potently suppress the differentiation of inducible T_reg_ cells [11], [12]. Taken together, these properties of IL-27 suggest it could enhance anti-tumor immunity by both promoting beneficial TH1 responses and suppressing T_reg_ activity.

Consistent with this proposition, a number of reports have suggested that IL-27 can promote anti-tumor cell line immune responses. These studies indicate IL-27 may act via a variety of mechanisms, including increasing IFN-γ production, promoting cytotoxic T cell and/or NK cell activity, inhibiting COX-2 expression or decreasing T_reg_ cell numbers [13], [14]. In each case, however, these studies have relied on transplanted cell line models and most have used forced expression of recombinant IL-27, or its receptor, ectopically in tumor cells lines [13]–[19]. In other words, previous work has been limited to studying late stage tumor cell expansion and excluded the processes of tumorigenesis and early stage neoplasia where immunosurveillance and immunoediting occur. Tumor cell lines, derived from established tumors, have already escaped host immunity in the originating mouse and have therefore already undergone cancer immunoediting. They are therefore likely to have different immunogenicity compared with tumors that develop *in situ*. Analysis of the effects of physiological IL-27 signaling on endogenously arising, heterogeneous tumors is required to properly assess the anti-tumor potential of this cytokine.

Here, we test the initiation and growth of carcinogen-induced fibrosarcomas and oncogene-driven mammary carcinomas in *Il27ra* deficient mice. Our data demonstrate that loss of IL-27 signaling leads to earlier emergence and more rapid growth of tumors. In addition, loss of IL-27 signaling results in reduced production of IFN-γ by CD4+ T cells and enhanced generation of regulatory T cells, effects which can inhibit an effective anti-tumor response. Our data therefore indicate a prominent role for IL-27 signaling in controlling physiologically arising tumors.

## Materials and Methods

### Ethics Statement

Mouse experimentation was carried out at the Garvan Institute of Medical Research and was handled in accordance with the Garvan Institute of Medical Research and St. Vincent's Hospital Animal Experimentation Ethics Committee, this study was specifically approved by that body (approval number 10/06).

### Mice

*Il27ra* ^−*/*−^ mice, *Il27ra^+/+^* mice [20] (C57BL/6 background, n>36) and C57BL6 mouse mammary epithelial cell (MMEC) donors were bred and housed in specific pathogen free conditions at the Garvan Institute of Medical Research. Adult mice (8–12 wk old) were used in all experiments.

### MCA-induced sarcoma model

Male *Il27ra^+/+^* and *Il27ra* ^−*/*−^ mice were inoculated s.c. in the right hind flank with a single dose of 100 μl of corn oil containing 25 μg or 10 μg of 3-methylcholanthrene (MCA) (Sigma-Aldrich). Mice were examined weekly for tumor development and subcutaneous tumor size were measured using calipers fitted with a vernier scale. Tumor diameter was calculated based on the average of two perpendicular measurements ([L+W]/2). When tumors reach >11 mm in diameter, mice were sacrificed and tumor tissues removed and processed for histological analysis.

### Polyoma middle T antigen (PyMT) driven-carcinoma model

PyMT expression was induced in primary MMEC isolated from C57BL6 mice by viral transduction of the pMIG-PyMT-IRES-GFP (pMIG-PyMT) construct as described previously [21], [22]. Infected MMECs (1.5×10^5^ cells in 10 μl PBS) were transplanted into the 4^th^ mammary fat pad of virgin female *Il27ra* ^+/+^ and *Il27ra* ^−/−^ mice. Tumor development was monitored by *in vivo* GFP imaging using the IVIS lumina II (Perkin Elmer) and measurement of palpable tumors for 280 days. For tumor transplant experiments, primary tumors were resected and cut into approx 1 mm^3^ pieces, which were surgically transplanted into the 4^th^ mammary fat pad of recipient female *Il27ra* ^+/+^ and *Il27ra* ^−*/*−^ mice.

### Flow cytometry

Single cell suspensions were made from spleens, tumor draining lymph nodes (TDLN; inguinal LN proximal to the tumor site), NDLN (contra-lateral inguinal LN) by mechanical disruption. Cells were stained for surface protein expression using the following Abs: anti-CD4 (RM4.5, BD), anti-CD8 (53-6.7, BD), anti-CD44 (IM7, eBioscience), anti-CD25 (PC61.5, eBioscience) and intracellularly stained with anti-FoxP3 (FJK-16s, eBioscience). Data for Foxp3+ cells is given as a percentage of CD4+CD25+ T cells. To assay *in vitro* cytokine production, cells were stimulated with PMA (50 ng/ml) and Ionomycin (500 ng/ml) for 5 hours with protein transport inhibitor (BD GolgiStop). Expression of cytokines was detected by intracellular staining using the following antibodies: anti-IFN-γ (XMG1.2, BD), anti-IL17 (17B7, eBioscience), anti-IL10 (JES5-16E3, Biolegend) and anti-IL4 (11B11, eBioscience), and data are given as a percentage of cytokine producing cells in the CD4+CD44+ gate.

### Immunohistochemistry

Paraffin embedded tumors were sectioned (5 μm) and stained using purified anti-mouse/rat FoxP3 (FJK-16s, eBioscience) and Biotin-SP conjugated F_ab_ goat anti-rat IgG (Jackson ImmunoResearch). Immunodetection was performed using Vectastain Elite ABC kit (Vector Laboratories) according to the recommendations of the manufacturer. T_reg_ cells were quantified by an observer, blinded to the genotype of the mice, by counting FoxP3+ cells in 5 independent fields of view (100× magnification) for each tumor section.

### Statistical Analysis

To determine the significance of differences in time to tumor incidence, a log-rank (Mantel-Cox) test was used. For comparing differences between genotypes at a single point in time, a two-tailed Student's t-test was used. P<0.05 was considered significant.

## Results

### Loss of IL-27 signaling leads to accelerated development of carcinogen-induced fibrosarcomas

To assess the role of endogenous IL-27 in tumor development, mice with a genetic deletion of the IL-27 receptor alpha chain (*Il27ra* ^−/−^) were used. These mice display no overt abnormalities and have been used extensively to study the effects of IL-27 on the immune system [9], [20], [23].

**Figure 1:**
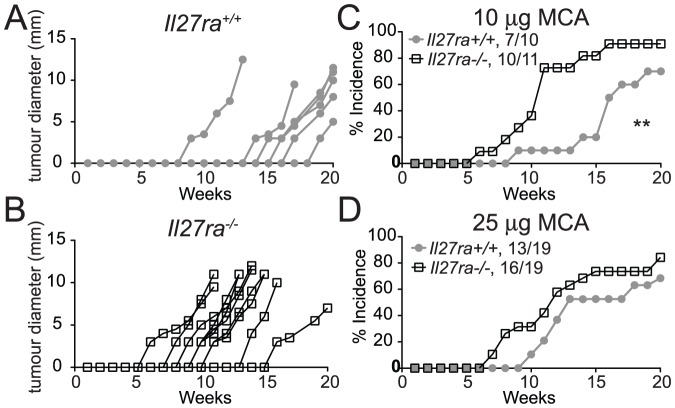
Rapid development of MCA-induced fibrosarcoma in *Il27ra*-deficient mice. Groups of *Il27ra* ^+/+^ (filled grey circles) and *Il27ra* ^−/−^ (open black squares) mice were treated with a single dose of 10 µg (A, B & C) or 25 µg (D) of MCA and tumor development was monitored weekly for 20 weeks. Tumors >3 mm in mean diameter and progressively growing were scored positive. (A & B) tumor growth curves of individual *Il27ra* ^+/+^ (A) and *Il27ra* ^−/−^ (B) mice with sarcoma. (C & D) cumulative incidence (%) in groups of mice injected with 10 μg (C; *Il27ra* ^+/+^ n = 10, *Il27ra* ^−/−^ n = 11, data are representative of 2 independent experiments) and 25 μg (D; data from 2 experiments are combined to give n = 19 per group) of MCA respectively. The overall tumor incidence is indicated. * p<.05 (Log-rank test).

Carcinogen-induced tumors allow analysis of spontaneous tumor initiation and growth without the introduction of new cells or genetic material. The 3-Methylcholanthrene (MCA)-induced fibrosarcama model has been used extensively to characterize immune-mediated control of tumor initiation and progression [1]. We therefore examined the effect of IL-27 signaling on MCA-induced fibrosarcoma development by administering MCA to groups of *Il27ra^+/+^* or *Il27ra* ^−*/*−^ mice. After injection of 10 μg MCA, palpable tumors were observed in 70% of *Il27ra^+/+^* controls (*n = 10*) and 90% of *Il27ra* ^−*/*−^ mice (*n = 11*) over the course of 20 weeks (Fig. 1 A–C). Tumor development occurred significantly earlier in *Il27ra* ^−*/*−^ mice, with median disease free survival of 11 weeks, compared with 16.5 weeks in *Il27ra^+/+^* mice (Fig. 1A–C). At a higher MCA dosage of 25 μg per mouse, 84% (*n = 19*) of *Il27ra* ^−/−^ mice developed sarcoma compared to 68% (*n = 19*) from *Il27ra^+/+^* mice. Again, sarcomas appeared slightly earlier in the *Il27ra* ^−/−^ mice (Fig 1D), although the difference did not reach statistical significance at this dose. Previous studies have also observed that the immune dependent protection is lost at higher doses of MCA [24]. These data show that IL-27 signaling plays a protective role, and point to important effects in the tumor initiation phase after carcinogen exposure.

### Loss of IL-27 signaling leads to accelerated development and growth of mammary carcinomas

Breast cancer is the most common cancer in women worldwide, comprising 16% of all female cancers (WHO). We therefore wished to test the effect of IL-27 signaling in a model of mammary carcinoma. A polyoma middle T (PyMT) induced mammary carcinoma model was used, wherein the expression of PyMT oncogene specifically in the mammary gland is achieved by retroviral transduction of primary mouse mammary epithelial cells (MMECs). Mammary neoplasias arise in this model through a process bearing close similarity with human breast cancer initiation [25].

**Figure 2:**
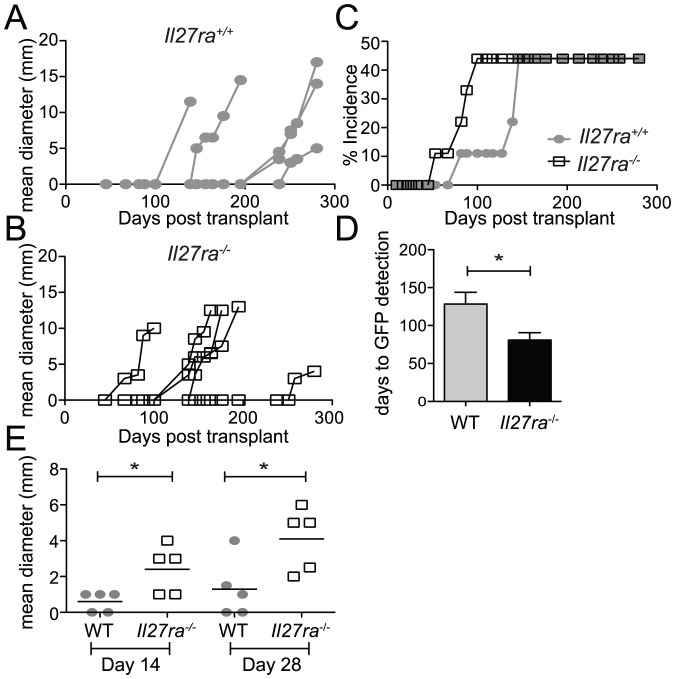
Accelerated development and growth of PyMT-induced mammary tumors in *Il27ra*-deficient mice. (A-D) WT mouse mammary epithelial cells (MMECs), retrovirally transduced with the pMIG-PyMT vector were transplanted to *Il27ra* ^+/+^ (filled grey circles) and *Il27ra* ^−/−^ (open black squares) hosts (n = 9/group). Tumor development and growth was monitored by *in vivo* GFP imaging and measurement of palpable tumors using Vernier calipers for 280 days. (A & B) tumor growth, as measured by calipers, of individual *Il27ra* ^+/+^ mice (A) and *Il27ra* ^−/−^ mice (B) with palpable mammary tumor. (C) cumulative % of mice with a detectable GFP signal in the mammary gland. (D) Average days to tumor detection (GFP) for mice that developed tumors for each genotype. (E) Primary mammary tumors that arose after implantation of pMIG-PyMT transduced tissue were excised and dissected into pieces (∼1 mm^3^) and surgically transplanted into groups of *Il27ra* ^+/+^ and *Il27ra* ^−/−^ mice (n = 5/group/time point). Mean tumor diameter (mm) at harvest, 14 and 28 days p.t., is shown. * p<.05 (unpaired t tests). Data presented are from one of three independent studies.

*Il27ra^+/+^* and *Il27ra* ^−/−^ mice were transplanted with primary C57BL6 MMECs transduced with pMIG-PyMT, encoding GFP as a reporter. Since we were interested in the kinetics of tumor initiation, we used the encoded GFP reporter to sensitively detect the emergence of tumors. In line with our observations in the carcinogen-induced model, palpable tumors (Fig. 2A,B) and GFP signal (Fig. 2 C,D and Fig. S1) were detected earlier in *Il27ra* ^−/−^ compared to *Il27ra^+/+^* mice. After 280 days, palpable tumor incidence in both *Il27ra^+/+^* and *Il27ra* ^−/−^ mice reached 55%. Amongst mice that developed tumors, the time to GFP detection was significantly earlier in the *Il27ra* ^−/−^ group (Fig. 2D). Two tumors, both arising late in the study, could not be detected by *in vivo* imaging. The lateness of detection may reflect that the tumors were deep in the tissue where GFP, having low tissue penetrance, was not detectable.

Due to the heterogeneity of mammary carcinoma appearance in this model (between 67 and 258 days p.t.), comparison of the immune phenotypes between the experimental groups could not be achieved at a common time point. We therefore tested the growth of established primary mammary carcinomas upon direct transfer to naive *Il27ra^+/+^* and *Il27ra* ^−/−^ hosts. Mice were sacrificed at days 14 and 28 days post-transplant (p.t.). Tumors implanted into *Il27ra* ^−/−^ grew significantly more rapidly compared to WT mice, with 4-fold and 3.15-fold larger tumors on average observed in *Il27ra* ^−/−^ mice at 14 and 28 days, respectively (Fig. 2E). This data show that IL-27 signaling plays an important role in protection against tumor growth in this mammary carcinoma model, as well as during carcinogen-induced tumorigenesis. Since IL-27 receptor is expressed predominantly on leukocytes and since IL-27 signaling is intact on the WT MMEC tumor tissue, enhanced tumor growth in *Il27ra* ^−/−^ mice can be attributed to reduced effectiveness of the anti-tumor immune response. The early tumor induction observed in *Il27ra* ^−/−^ mice in both cancer models suggests that IL-27 signals play an important role in immunosurveillance during neoplasia. Accelerated growth of established tumor tissue in the mammary tumor transplant model (Fig. 2E) suggests that IL-27 signals can contribute to anti-tumor responses during carcinoma growth, as well as influencing tumor initiation.

### Reduced IFN-γ production in tumor bearing *Il27ra* ^−*/*−^ mice

In order to compare the immune responses occurring in *Il27ra^+/+^* and *Il27ra* ^−*/*−^ animals, it is necessary to choose a common time point. The rapid emergence of MCA-induced tumors in *Il27ra* ^−*/*−^ mice, and subsequent euthanasia, meant that a suitable time point could not be found using a 10 µg dose of MCA. We therefore examined the immune phenotype of mice injected with 25 µg MCA at 14 weeks post-induction, a time at which the majority of mice injected had developed palpable sarcomas (Fig. 1D). To overcome the kinetic heterogeneity of primary PyMT driven mammary carcinoma development (Fig. 2 A–B), transplantation of primary tumor into recipient mice (Fig. 2E) was used for analysis of the immune phenotype.

To examine the immunological effects of the loss of IL-27 signaling during the anti-tumor response, we isolated TDLN, NDLN and spleens from tumor bearing *Il27ra^+/+^* and *Il27ra* ^−/−^ mice. A panel of immune cell subset markers was assessed by flow cytometry. No significant differences were observed in the total percentages or cell numbers of naïve, effector or central memory CD4 or CD8 T cells (based on CD62L and CD44 expression; NK cells (NK1.1^+^TCRβ^−^); NKT cells (CD1d-tetramer^+^TCRβ^+^), CD11c^+^, CD11b^+^ or GR1^+^ granulocytes, nor myeloid suppressor cells (CD11b^+^ GR1^+^)(d.n.s.).

**Figure 3:**
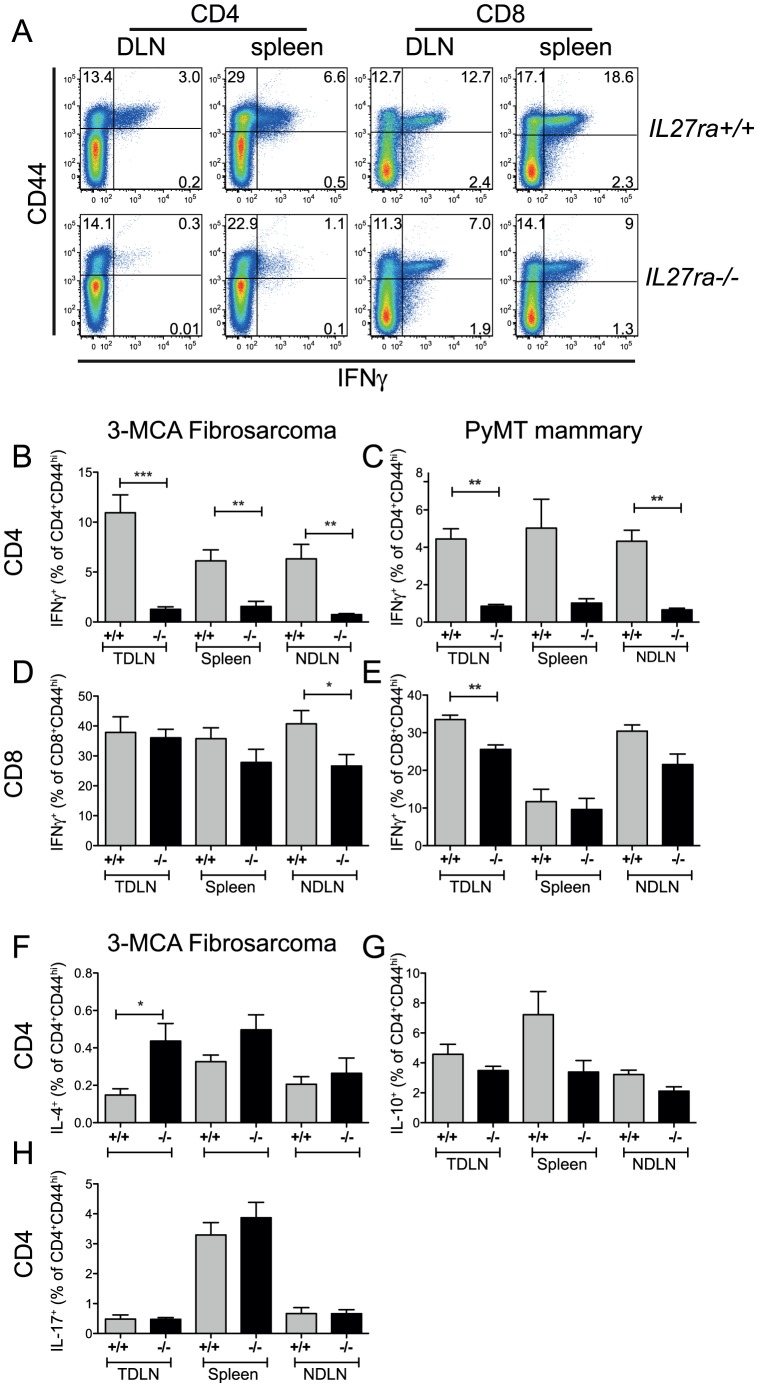
Reduced IFN-γ production by activated CD4^+^ T cells in tumor bearing *Il27ra*-deficient mice. (A, B, D, F–H) Fibrosarcomas were induced in *Il27ra* ^+/+^ (grey bars) and *Il27ra* ^−/−^ (black bars) by injecting 25 µg of MCA s.c.. 14 wk after tumor induction, lymphocytes from spleen, tumor draining LN (TDLN; inguinal) and non tumor draining LN (NDLN; contralateral inguinal) of tumor bearing mice were harvested (n = 6 per genotype). (C, E) PyMT-driven mammary carcinoma cells were transplanted into *Il27ra* ^+/+^ (grey bars) and *Il27ra* ^−/−^ (black bars) mice. 32 days after tumor transplantation, lymphocytes from spleen, TDLN and NDLN of tumor bearing mice were harvested (n = 3 per genotype). Cells were re-stimulated with PMA and ionomycin for 5 hours in the presence of a protein transport inhibitor then IFNγ, IL-4, IL-10 and IL-17 production was determined by flow cytometry. (A) Representative cytometry plots illustrating IFNγ versus CD44 expression. (B-H) The percentage of cytokine (as indicated) producing cells in the CD4^+^ CD44^hi^ gate (B, C, F-H) or the CD8^+^ CD44^hi^ gate (D, E) is shown. Error bars indicate SEM. * p<.05, ** p<.01, *** p<.001 (unpaired t tests). Each dataset is representative of two independent studies.

Cytokine production by T cells from *Il27ra^+/+^* and *Il27ra* ^−/−^ tumor bearing mice was assessed by flow cytometry. Since the tumor antigens are not known in these spontaneously arising tumors, PMA and Ionomycin were used to polyclonally restimulate previously activated T cells. IFN-γ production was found to be significantly reduced in the CD4^+^CD44^+^ T cell compartment in *Il27ra* ^−/−^ compared to *Il27ra^+/+^* mice in both the fibrosarcoma (Fig. 3A, B) and mammary carcinoma models (Fig. 3C). The caveat of polyclonal restimunation is that non-specific antigen experienced cells were also activated. However, increased percentages of total CD44^+^ T cells observed in the TDLN compared with NDLN (d.n.s) suggest that tumor specific responses were occurring. This data are in line with previous reports that IL-27 can promote TH1 responses and that IFN-γ levels are reduced in *Il27ra* ^−/−^ mice during certain immunological challenges [26]–[29]. Decreased percentages of IFN-γ producing cells were also observed in the CD8^+^ compartment of *Il27ra* ^−/−^ mice, although the magnitude of this reduction was diminished compared with the CD4 compartment (Fig. 3 D,E).

IL-27 has also been shown to enhance IL-10 production and negatively regulate IL-4 and IL-17 [5], [10], however, subtle effects of *Il27ra* genotype were observed for these cytokines in the tumor models. The TH2 associated cytokine IL-4 was found to be produced by a significantly higher proportion of *Il27ra* ^−/−^ CD4^+^ T cells in the TDLN, and numbers of splenic IL-10 producing cells were significantly reduced, when MCA-induced fibrosarcoma bearing mice were analyzed (Fig. 3 F,G), while IL-17 producing CD4+ T cells remained unchanged (Fig 3H). Thus the major difference in cytokine expression appears to be in the IFN-γ production by the CD4^+^ T helper subset.

### Increased T_reg_ cells in tumor tissue and lymphoid organs of *Il27ra* ^−*/*−^ mice

T_reg_ cells are CD4^+^ T cells that actively suppress the function of effector T cells, and are usually defined based on expression of the transcription factor Foxp3. In the MCA fibrosarcoma model, T_reg_ cells have been demonstrated to suppress the anti-tumor response, allowing accelerated tumor growth [30], [31]. We have recently shown that *Il27ra* ^−/−^ CD4^+^ T cells show a greater propensity to differentiate into T_reg_ cells after activation [11]. We therefore investigated T_reg_ cells in tumor bearing mice.

**Figure 4:**
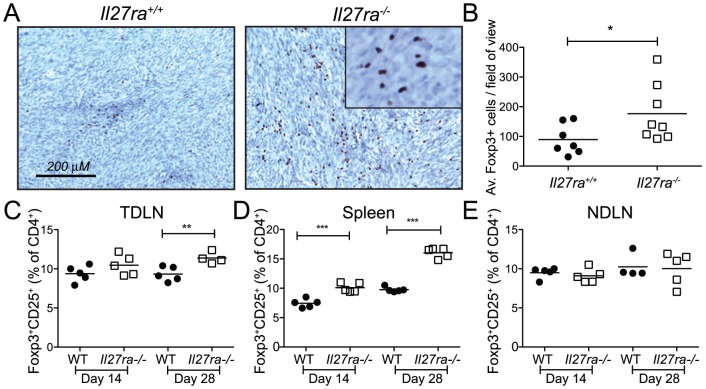
Increased percentage of T_reg_ cells in *Il27ra*-deficient mice. (A & B) tumors induced by injection of 25 µg of MCA into *Il27ra* ^+/+^ and *Il27ra* ^−/−^ were harvested at 14 weeks post-induction and 5 µm sections analyzed by immunohistochemistry to detect FoxP3^+^ cells. (A) Representative photographs (magnification = 100×) of tumor sections stained with FoxP3 (brown). (B) The average Foxp3^+^ cells per field of view, over 5 fields of view per mouse, is indicated (n = 7 per group). Bars indicate the average of 7/8 mice per genotype. (C-E) 14 or 28 days after PyMT tumor transplant (as indicated), lymphocytes from *Il27ra* ^+/+^ (filled grey circles) and *Il27ra* ^−/−^ (open black squares) host spleen, TDLN and NDLN were harvested. Single cell preparations were stained with antibodies against CD4, CD25 and Foxp3 for flow cytometry. The percentage of FoxP3^+^CD25^+^ cells in the CD4^+^ compartment is shown for (C) TDLN, (D) spleen and (E) non-tumor draining lymph node. n = 5 per group *p<.05, **p<.01, ***p<.001 (unpaired t test).

Immunohistochemical analysis of FoxP3^+^ cells within MCA tumor tissues revealed an average 2-fold increased T_reg_ numbers in *Il27ra* ^−/−^ compared to *Il27ra^+/+^* mice (Fig. 4 A,B). In addition, significantly increased T_reg_ cell percentages were observed in TDLN (day 28; Fig. 4C) and in the spleen (Fig. 4D) of *Il27ra* ^−/−^ mice with PyMT tumor transplants, compared to *Il27ra^+/+^* controls. The lack of an observed difference in T_reg_ numbers in the non-draining LN (Fig. 4E), distant from the immune activation site, as well as the observation that T_reg_ percentages are not altered in unchallenged or mock-transplanted *Il27ra* ^−/−^ mice ([11] and d.n.s.) suggest that changes in T_reg_ percentage are in direct response to the tumor challenge. These data indicate that T_reg_ numbers are controlled by IL-27 signaling in tumor tissues and peripheral lymphoid organs during anti-tumor immune responses. This finding is consistent with the observed increase in tumor growth and with our previous work showing that *Il27ra* ^−/−^ T cells more readily differentiate into T_reg_ [11].

## Discussion

Our data demonstrate that IL-27 signaling enhances the anti-tumor immune response during *in vivo* development and growth of two diverse tumor types. MCA-induced fibrosarcoma and PyMT-driven mammary carcinoma development are accelerated in *Il27ra*-deficient mice and are accompanied by reduced IFN-γ production and increased percentages (and numbers) of T_reg_ cells.

The importance of investigating the role of cytokines in a physiological context is illustrated in the case of IL-27 family member IL-23. Strategies testing rIL-23 overexpression in tumor cells resulted in enhanced anti-tumor responses and inhibited cancer progression, analogous to IL-12 and IL-27 [17]. However, studies involving carcinogen-induced tumors (TPA-induced skin papillomas) or syngenic tumor transplants into IL-23 deficient mice clearly showed that they were resistant to tumor development, leading to the current consensus, that IL-23 is in fact pro-tumorigenic [32], [33]. Moreover, studying tumors that arise *in situ* is necessary to understand the process of immunosurveillance. Established tumor derived cell lines have already escaped host immunity and are therefore likely to have pre-altered immunogenicity. Thus, analysis on the role of physiological IL-27 signaling on endogenously arising tumors is paramount to properly assess the anti-tumor potential of this cytokine.

The MCA-induced fibrosarcoma model has been used extensively to characterize immune-mediated control of tumor initiation and progression [1]. Many immune mediators have been demonstrated to influence tumor development and progression in this model. IL-27 protein family members IL-12 and IL-23 have been shown to have important, although opposing, effects on tumor development in this model [33], [34]. It is known that loss of TH1 associated signals, such as IFN-γ, IFN receptors, STAT-1, and IL-12p40, lead to increased susceptibility to MCA induced fibrosarcomas. Recently, T_reg_ cells have been shown to have a deleterious effect on the response to these tumors, with T_reg_ depletion resulting in reduced tumor development and even eradication of some established tumors [30], [31]. Thus, the loss of IFN-γ production and the increased development of T_reg_ cells in Il27ra deficient mice fits well with rapid tumor development observed. NK, NKT and CD8+ T cells are also known to be important participants in immune mediated protection against MCA induced tumorigenesis [1], however, we did not observe significant changes in these populations in tumor bearing *Il27ra* ^−/−^ mice.

The immunological control of tumorigenesis in PyMT-driven mammary carcinomas is less well defined. However, modulation of cytokine and chemokine expression in PyMT transgenic mice has been shown to alter tumor growth, suggesting that host immune responses are important in determining disease outcome in this model [35], [36]. The accelerated development and growth of PyMT driven mammary carcinomas in *Il27ra* ^−/−^ mice confirms the immunological contribution in this model and recapitulate the data from the MCA fibrosarcomas, wherein tumor bearing *Il27ra* ^−/−^ mice have reduced IFN-γ and increased T_reg_ cells.

Together, these two models provide strong evidence for an important role for IL-27 signals in promoting anti-tumor immunity against *de novo* tumors. The early tumor induction observed in *Il27ra* ^−/−^ mice in both cancer models suggests that IL-27 signals play an important role in immunosurveillance during neoplasia. Our observation of accelerated growth of established primary mammary carcinomas, as well as a previous report that tumor cell lines grow more rapidly in *Il27ra* ^−/−^ mice [37], show that IL-27 mediated enhancement of the immune response also limits late stage tumor growth.

This study is the first to show that physiological IL-27 signaling plays a protective role in immunity against autochthonous tumors. Our results are in agreement with a previous study that showed more rapid growth of B16F10 cell tumors in *Il27ra* ^−/−^ mice, compared with WT controls [37]. Our data also concur with studies that used IL-27-transduced cancer cell lines, including colon carcinoma [16], TBJ neuroblastoma [14], [38] and B16F10 melanoma [17], [18], [37], [39], which showed that induced IL-27 expression, from tumor tissue itself, enhanced the protective immune response.

Effective anti-tumor immune responses are associated with a TH1 response and ergo, high IFN-γ levels. Anti-tumor effects of IFN-γ include induction of anti-angiogenic factors IP-10 and MIG, upregulation of MHC class I on tumor cells, sensitization of tumor cells to apoptosis and enhancing CTL and NKT cell activity [2], [40]. IL-27 can clearly promote TH1 activity *in vitro* through the phosphorylation of STAT-1, and upregulation of T-bet and IL-12Rβ2 [41], [42]. However, whether or not IL-27 is required for IFN-γ production by CD4+ T cells *in vivo* has been controversial. IL-27 signals were required for IFN-γ production during DSS colitis, while loss of *Il27ra* resulted in a transient defect in IFN-γ production in some models infectious models, for example, *Leishmania major* [29]. Other infectious challenges, such as *Mycobacterium tuberculosis, Toxoplasma gondii* and *Leishmania donovani* elicited strong TH1 responses in *Il27ra* ^−*/*−^ mice (reviewed in [5]). Thus, the necessity for IL-27 appears to be context dependent. Our data suggest that IL-27 signals are indispensable for IFN-γ production by CD4+ T cells in tumor immunity.

The moderate reduction in IFN-γ production by CD8+ cells in *Il27ra* ^−*/*−^ mice in tumor models is in contrast with infectious models, where a strong requirement for IL-27R signaling and T-bet induction was observed [43]. These data suggest that other T-bet inducing cytokines may be able to compensate in CD8+ T cells for the loss of IL-27 signals in tumor models.

Our observation of increased T_reg_ cells in *Il27ra* ^−*/*−^ mice is also in line with the known function of IL-27. We have recently reported that IL-27 suppresses the development of inducible Foxp3+ T_reg_ cells *in vivo* [11] and IL-27 is known to antagonize TGFβ driven T_reg_ differentiation *in vitro* [12], [44]. We and others have also previously reported reduced IL-10 [45]–[48], increased IL-17 [23], [49] and increased IL-4 [26] in *Il27ra* ^−*/*−^ mice. While the observed changes in these cytokines in tumor bearing mice were consistent with previous reports, the magnitude of the changes was small compared with IFN-γ and was not significant across all the tissues examined, suggesting minor roles in the IL-27 mechanism of action during the anti-tumor response.

In summary, these data demonstrate the physiological importance of IL-27 signals during immunosurveillance and anti-tumor responses in mouse models of cancer. Together with a recent report that reduced serum IL-27 level is a risk factor for esophageal cancer [50], our data support the concept that IL-27 signaling agonists could be beneficial in immunotherapy for the treatment of cancer.

## Supporting Information

Figure S1 **Representative in vivo GFP imaging using the IVIS lumina II to detect pMIG-PyMT (GFP) vector transduced cell expansion.** 3/9 from the Il27ra-/- mice showing positive signal compared to 1/9 from the Il27ra+/+ mice 88 days post-transplant.
(EPS)
